# Supplementary material for: Stabilization of mineral-associated organic carbon in Pleistocene permafrost
Source: Nat Commun. 2023 Apr 13;14:2120. doi: 10.1038/s41467-023-37766-5 (PMC10102184; doi:10.1038/s41467-023-37766-5)
Supplement: Supplementary file 1 — Supplementary information [file 41467_2023_37766_MOESM1_ESM.pdf]

Supplementary Information

Stabilization of mineral-associated organic carbon in Pleistocene permafrost

Jannik Martens[1,2], Carsten W. Mueller[3,4], Prachi Joshi[5], Christoph Rosinger[6,7,8], Markus Maisch[5], Andreas Kappler[5,9], Michael Bonkowski[6], Georg Schwamborn[10,11], Lutz Schirrmeister[10] and Janet Rethemeyer[1]

[1]Institute of Geology and Mineralogy, University of Cologne, Cologne, Germany
[2]Lamont-Doherty Earth Observatory, Columbia University, New York, USA
[3]Chair for Soil Science, Technical University of Munich, Freising, Germany
[4]Department of Geosciences and Natural Resource Management, University of Copenhagen, Copenhagen, Denmark
[5]Department of Geosciences, University of Tübingen, Tübingen, Germany
[6]Institute of Zoology, University of Cologne, Cologne, Germany
[7]Institute of Agronomy, University of Natural Resources and Life Sciences, Tulln an der Donau, Austria
[8]Institute of Soil Research, University of Natural Resources and Life Sciences, Vienna, Austria
[9]Cluster of Excellence: EXC 2124: Controlling Microbes to Fight Infection, Tübingen, Germany
[10]Alfred-Wegener-Institute Helmholtz Centre for Polar and Marine Research, Permafrost Research Section, Potsdam, Germany
[11]Eurasia Institute of Earth Sciences, Istanbul Technical University Maslak, Istanbul, Turkey

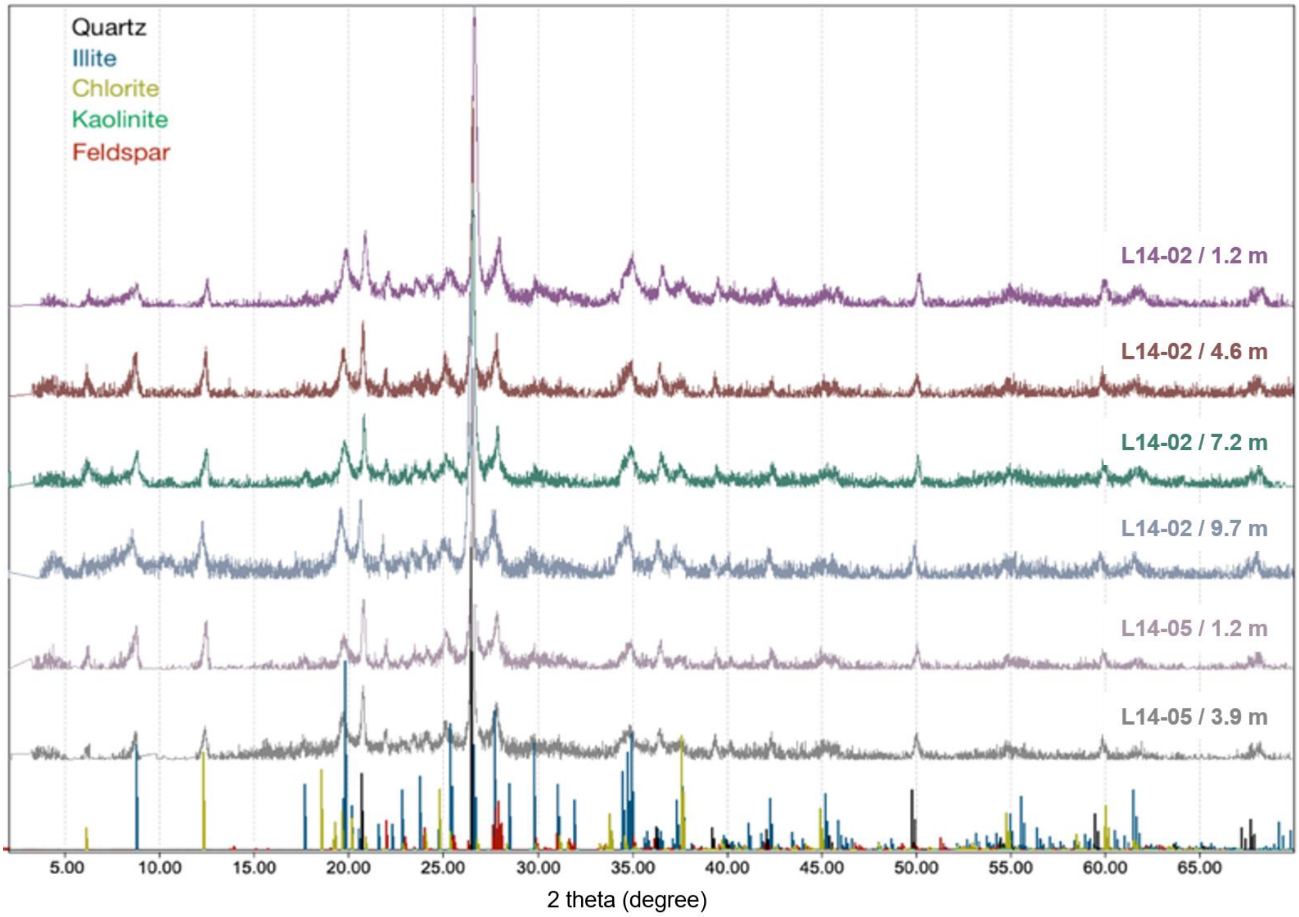

Supplementary Figure 1: X-ray diffraction patterns of mineral-associated organic matter <6.3 µm ($MAOM_{<6.3µm}$) fractions of samples from Yedoma deposits (L14-02) and post-glacial thermokarst sediments (L14-05).

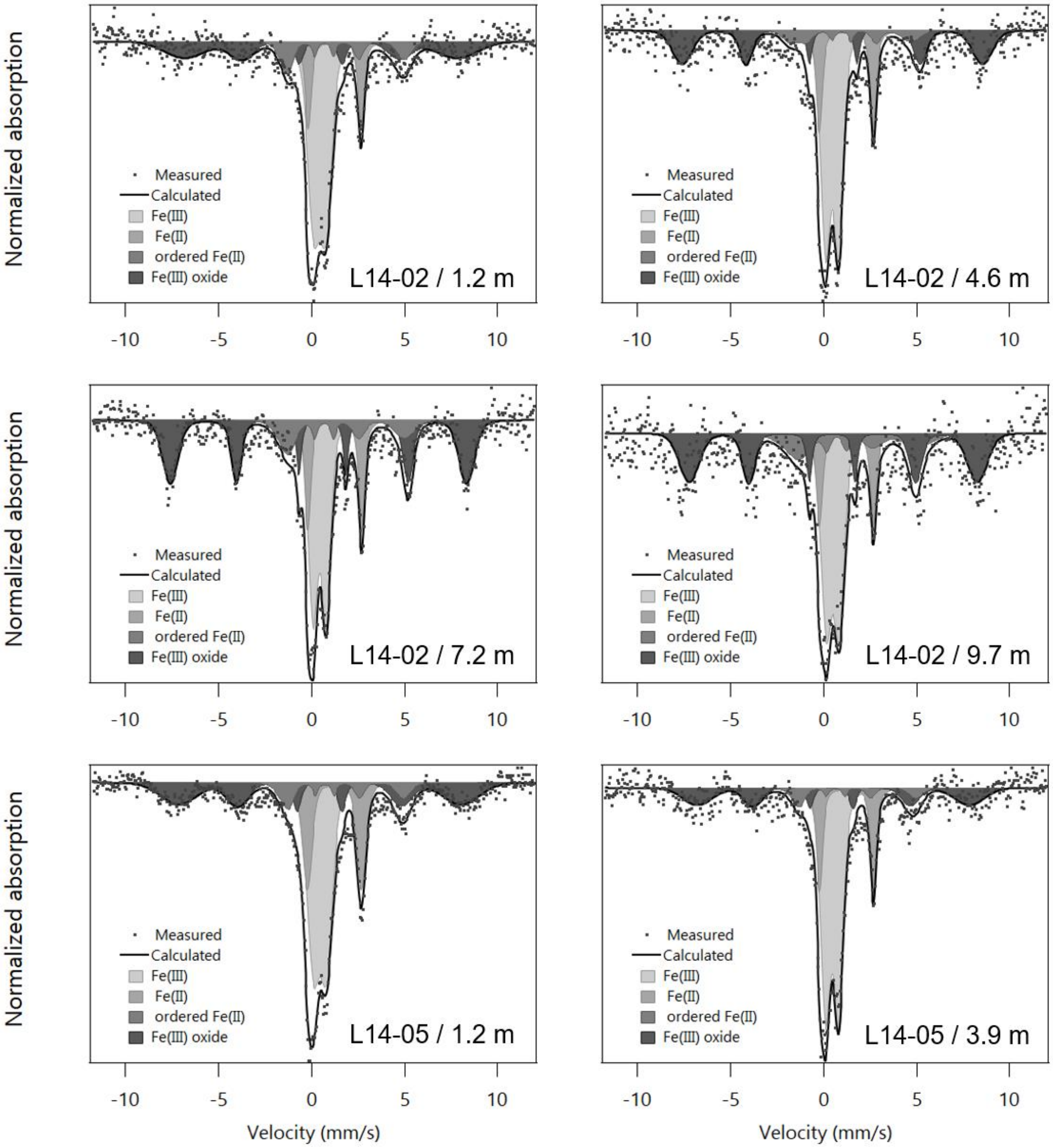

Supplementary Figure 2: Mössbauer spectroscopy analysis of iron (Fe) mineral phases collected at 5K of mineral-associated organic matter <6.3 µm ($MAOM_{<6.3µm}$) fractions of samples from Yedoma deposits (L14-02) and post-glacial thermokarst sediments (L14-05).

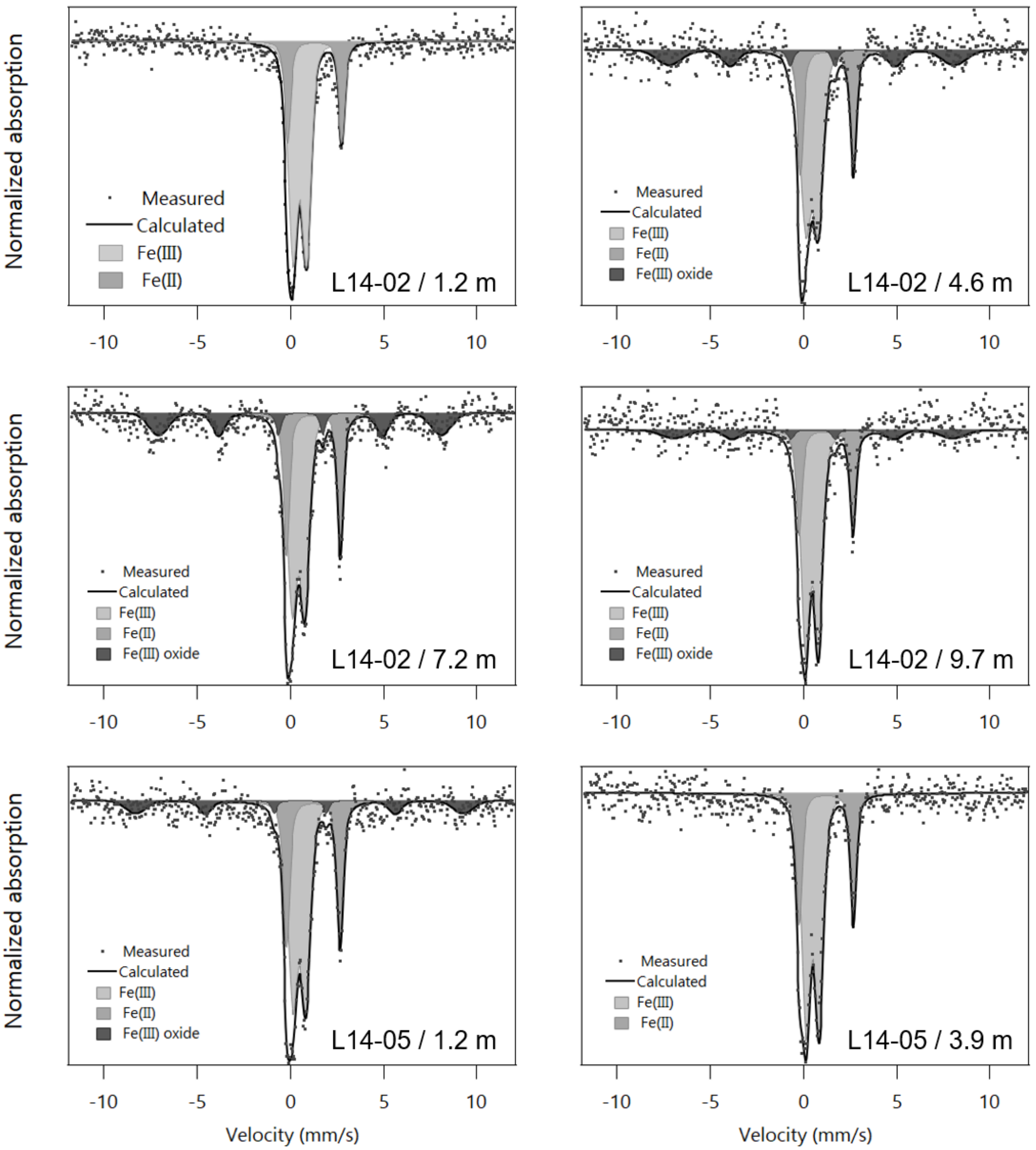

Supplementary Figure 3: Mössbauer spectroscopy analysis of iron (Fe) mineral phases collected at 77K of mineral-associated organic matter <6.3 µm ($MAOM_{<6.3\mu m}$) fractions of samples from Yedoma deposits (L14-02) and post-glacial thermokarst sediments (L14-05).

Supplementary Table 1: Content and composition of organic carbon (OC), OC/N, *n*-alkanes and the carbon preference index (CPI) of *n*-alkanes in bulk sediments of post-glacial thermokarst sediments (15-11 ka BP) in cores L14-05 and Yedoma deposits in L14-02 (55-33 ka BP).

**Core No**	**Depth**<br>m b.s.	**Age**<br>ka BP	**OC**<br>mg OC g^{-1} bulk-wt	**OC/N**	***n*-alkanes C_{25},C_{27},C_{29}**<br>µg g^{-1} OC	**CPI_{alk}**
L14-05	1.2	15-11	4.4	8.8	878	4.8
	3.9	15-11	15.5	8.8	560	5.0
L14-02	1.2	48-33	47.3	12.7	230	6.9
	4.6	48-33	25.5	12.0	192	6.4
	7.2	55-48	12.5	11.9	421	4.8
	9.7	55-48	14.2	10.8	11	1.9

Supplementary Table 2: Distribution and composition of soil fractions in weight% of the bulk dry weight (DW), as well as the organic carbon (OC) content and OC/N ratios, including free (fPOM), occluded particulate organic matter ($oPOM_{<20µm}$) and mineral-associated organic matter ($MAOM_{<6.3µm}$).

**Parameter**	**Core No**	**Depth** (m b.s.)	**fPOM**	**$oPOM_{<20µm}$**	**63 - 200** (µm)	**20 - 63** (µm)	**6.3 - 20** (µm)	**$MAOM_{<6.3µm}$** (µm)
wt% of bulk DW	L14-05	1.2	0.4	0.1	0.3	58.7	30.1	10.4
		3.9	0.2	0.6	0.0	10.1	43.6	45.3
	L14-02	1.2	22.2	2.7	0.4	21.7	24.8	27.5
		4.6	0.8	1.9	1.9	29.6	29.7	35.8
		7.2	0.3	0.6	0.7	45.1	25.1	28.0
		9.7	0.7	0.5	0.6	30.0	32.8	35.0
OC mg OC g^{-1} fraction DW	L14-05	1.2	38.8	353.9	3.2	0.6	0.8	19.5
		3.9	102.0	358.3	73.8	0.9	2.9	26.0
	L14-02	1.2	155.3	310.1	2.5	5.9	11.8	58.6
		4.6	286.0	319.5	3.2	1.6	3.5	26.5
		7.2	168.2	346.6	2.4	1.2	1.8	25.0
		9.7	107.1	331.3	2.0	1.2	1.9	30.1
OC/N	L14-05	1.2	16.2	16.0	23.5	5.2	10.5	7.3
		3.9	14.6	15.8	17.8	6.8	7.0	7.3
	L14-02	1.2	15.2	14.2	17.3	12.2	10.9	9.3
		4.6	19.6	13.9	24.0	18.7	6.4	10.7
		7.2	14.4	14.5	nd	11.6	13.0	8.1
		9.7	15.1	14.5	16.8	9.1	4.4	8.4

Supplementary Table 3: Radiocarbon (^{14}C) contents and conventional ages (in years before present – BP) of the bulk sedimentary organic matter in subsamples taken from cores L14-02 and L14-05, of free (fPOM) and occluded particulate organic matter ($oPOM_{<20µm}$), and mineral-associated organic matter ($MAOM_{<6.3µm}$) from these samples.

**Core**	**depth** (m b.s.)	**fraction**	**^{14}C age** (yr BP)	**$\Delta^{14}C$** (‰ ± 1σ)	**Cologne AMS sample label**
L14-05	1.2	bulk	16500 ± 90	-872.8 ± 9.3	COL3684.1.1
		fPOM	13050 ± 60	-804.5 ± 6.2	COL3681.1.1
		$oPOM_{<20µm}$	16300 ± 80	-869.6 ± 9	COL3682.1.1
		$MAOM_{<6.3µm}$	15460 ± 80	-855.1 ± 8.3	COL3683.1.1
L14-05	3.9	bulk	18190 ± 100	-896.9 ± 11.3	COL3688.1.1
		fPOM	15980 ± 60	-864.2 ± 6.9	COL3685.1.1
		$oPOM_{<20µm}$	16550 ± 90	-873.6 ± 9.5	COL3686.1.1
		$MAOM_{<6.3µm}$	17960 ± 90	-893.9 ± 10.1	COL3687.1.1
L14-02	1.2	bulk	33400 ± 230	-984.5 ± 28.3	COL3672.1.1
		fPOM	32500 ± 210	-982.6 ± 26.1	COL3669.1.1
		$oPOM_{<20µm}$	32500 ± 220	-982.6 ± 27.1	COL3670.1.1
		$MAOM_{<6.3µm}$	32700 ± 220	-983.1 ± 27.2	COL3671.1.1
L14-02	4.6	bulk	44800 ± 720	-996.3 ± 88.8	COL3675.1.2
		fPOM	38700 ± 310	-992.0 ± 38.2	COL3673.1.1
		$oPOM_{<20µm}$	40800 ± 350	-993.8 ± 42.7	COL3674.1.1
		$MAOM_{<6.3µm}$	40400 ± 320	-993.5 ± 39.9	COL3676.1.2
L14-02	7.2	bulk	>54000	-999.0 ± 28	COL3680.1.1
		fPOM	23400 ± 130	-946.0 ± 14.8	COL3677.1.1
		$oPOM_{<20µm}$	38700 ± 290	-992.0 ± 36	COL3678.1.1
		$MAOM_{<6.3µm}$	36900 ± 340	-989.9 ± 41.9	COL3679.1.1
L14-02	9.7	bulk	>54000	-999.4 ± 45.8	COL3696.1.1
		fPOM	36100 ± 450	-989.0 ± 55.8	COL3693.1.1
		$oPOM_{<20µm}$	34300 ± 240	-986.2 ± 29.7	COL3694.1.1
		$MAOM_{<6.3µm}$	40500 ± 480	-993.6 ± 59.5	COL3695.1.1

Supplementary Table 4: Relative signal intensities and ratio of compound classes identified with ^{13}C-CPMAS NMR spectroscopy of free (fPOM) and occluded particulate organic matter ($oPOM_{<20µm}$) and mineral-associated organic matter ($MAOM_{<6.3µm}$) fractions of permafrost cores L14-02 and L14-05.

Core	Depth	Fraction	alkyl	O/N-alkyl	aryl	carboxyl	Alkyl/ O/N-alkyl
	m b.s.		%	%	%	%	
L14-05	1.2	$MAOM_{<6.3µm}$	47.2	25.7	12.2	13.2	1.8
	1.2	$oPOM_{<20µm}$	36.7	39.5	15.8	8.2	0.9
	1.2	fPOM	31.1	29.9	23.3	13.6	1.0
	3.9	$MAOM_{<6.3µm}$	41.0	21.0	17.2	18.6	2.0
	3.9	$oPOM_{<20µm}$	24.8	37.6	25.9	11.2	0.7
	3.9	fPOM	27.1	37.5	25.4	9.3	0.7
L14-02	1.2	$MAOM_{<6.3µm}$	21.9	24.0	30.1	21.8	0.9
	1.2	$oPOM_{<20µm}$	22.4	60.7	11.9	4.8	0.4
	1.2	fPOM	34.2	46.2	9.9	8.8	0.7
	4.6	$MAOM_{<6.3µm}$	31.0	38.5	15.2	14.5	0.8
	4.6	$oPOM_{<20µm}$	18.1	56.0	18.4	7.3	0.3
	4.6	fPOM	15.6	57.4	17.0	9.5	0.3
	7.2	$MAOM_{<6.3µm}$	23.7	29.6	22.4	22.1	0.8
	7.2	$oPOM_{<20µm}$	26.1	41.3	18.9	12.9	0.6
	7.2	fPOM	n.d.	n.d.	n.d.	n.d.	n.d.
	9.7	$MAOM_{<6.3µm}$	25.1	34.5	20.8	18.5	0.7
	9.7	$oPOM_{<20µm}$	20.2	52.1	19.4	8.2	0.4
	9.7	fPOM	n.d.	n.d.	n.d.	n.d.	n.d.

Supplementary Table 5: Absolute concentrations of individual *n*-alkanes in µg g^{-1} OC.

Core	Depth (m b.s.)	C21	C22	C23	C24	C25	C26	C27	C28	C29	C30	C31	C32	C33
L14-05	1.2	99	86	198	83	224	60	307	54	347	31	355	20	135
	3.9	50	44	97	43	119	36	191	43	250	26	291	25	122
L14-02	1.2	16	10	30	11	43	11	87	14	100	8	115	5	28
	4.6	7	7	18	9	31	10	68	16	93	7	101	5	35
	7.2	27	29	65	30	90	28	148	31	183	16	145	9	56
	9.7	11	11	16	5	6	1	3	0	2	0	2	0	1

Supplementary Table 6: Relative spectral areas (%) of iron (Fe) mineral phases in mineral-associated organic matter ($MAOM_{<6.3 \mu m}$) fractions in permafrost cores L14-05 and L14-02 based on ^{57}Fe Mössbauer spectroscopy.

**Core**	**Depth**	**OC/Fe**	**Fe(II)**	**Fe(III)**	**Fe(III) oxide**	**Fe(II)**	**Fe(III)**	**Ordered[1] Fe(II)**	**Fe(III) oxide**	**Fe(III)/ Fe(II)**
	(m b.s.)	wt/wt	77K	77K	77K	5K	5K	5K	5K	77K
L14-05	1.2	1.1	30.9	55.1	14.0	20.7	42.6	8.6	28.1	2.9
	3.9	0.6	30.2	69.8		17.5	53.2	7.0	22.3	2.9
L14-02	1.2	0.5	25.8	74.2		13.8	47.8	11.3	27.1	2.7
	4.6	0.7	25.9	53.4	20.7	16.2	49.5	13.9	20.5	3.2
	7.2	0.5	26.8	50.4	22.8	14.6	35.6	12.9	36.9	2.2
	9.7	0.5	23.7	63.6	12.6	12.2	38.3	14.6	34.8	2.3

[1]magnetically ordered Fe(II) at 5 K (full spectra are shown in Supplementary Figure 2 and 3)

Supplementary Table 7: Basal respiration measurements (mean±s.d.) of mineral-associated organic matter ($MAOM_{<6.3μm}$) fractions in permafrost cores L14-05 and L14-02.

Core	Depth (m b.s.)	Sample weight (g)	Time interval (hrs)	Basal respiration (µg CO_2 g^{-1} dw h^{-1})	Basal respiration (µg CO_2 g^{-1} OC h^{-1})
L14-05	1.2	1.5	70-114	3.33 ± 0.21	170.8 ± 11
	3.9	1.5	50-100	2.07 ± 0.27	79.4 ± 11
L14-02	1.2	1.5	90-114	2.29 ± 0.29	39.1 ± 3
	4.6	1.5	50-100	1.85 ± 0.35	69.7 ± 13
	7.2	1.5	70-114	1.33 ± 0.19	53.5 ± 8
	9.7	1.5	40-100	1.64 ± 0.21	54.4 ± 7

Supplementary Table 8: Estimation of organic carbon stocks associated with mineral-associated organic matter ($MAOM_{<6.3\mu m}$) material, as well as free and occluded particulate OM (fPOM; $oPOM_{<20\mu m}$). The calculation is based on published estimates of OC stocks in Yedoma deposits and thermokarst sediments[1].

**Yedoma deposit[1]**	**Thermokarst sediment[1]**	**Yedoma deposits 55-33 ka (this study)**		**Thermokarst sediment 15-11 ka (this study)**		**Sum**
(Gt)	(Gt)	fraction of bulk	Gt	fraction of	Gt	Gt
		$MAOM_{<6.3\mu m}$				
83 ± 12	130 ± 29	0.49 ± 0.18	40 ± 21	0.59 ± 0.20	77 ± 44	117 ± 65
		fPOM				
83 ± 12	130 ± 29	0.22 ± 0.32	18 ± 29	0.02 ± 0.01	3 ± 3	21 ± 32
		$oPOM_{<20\mu m}$				
83 ± 12	130 ± 29	0.17 ± 0.05	14 ± 6	0.11 ± 0.04	14 ± 8	28 ± 14

[1]Stock estimates based on ref.[2]
